# Plasmid-Mediated Bioaugmentation for the Bioremediation of Contaminated Soils

**DOI:** 10.3389/fmicb.2017.01966

**Published:** 2017-10-09

**Authors:** Carlos Garbisu, Olatz Garaiyurrebaso, Lur Epelde, Elisabeth Grohmann, Itziar Alkorta

**Affiliations:** ^1^Soil Microbial Ecology Group, Department of Conservation of Natural Resources, Neiker Tecnalia, Derio, Spain; ^2^Instituto Biofisika (UPV/EHU, CSIC), Department of Biochemistry and Molecular Biology, University of the Basque Country, Bilbao, Spain; ^3^Beuth University of Applied Sciences, Berlin, Germany

**Keywords:** biodegradation, catabolic plasmid, fitness cost, horizontal gene transfer, soil pollution

## Abstract

Bioaugmentation, or the inoculation of microorganisms (e.g., bacteria harboring the required catabolic genes) into soil to enhance the rate of contaminant degradation, has great potential for the bioremediation of soils contaminated with organic compounds. Regrettably, cell bioaugmentation frequently turns into an unsuccessful initiative, owing to the rapid decrease of bacterial viability and abundance after inoculation, as well as the limited dispersal of the inoculated bacteria in the soil matrix. Genes that encode the degradation of organic compounds are often located on plasmids and, consequently, they can be spread by horizontal gene transfer into well-established, ecologically competitive, indigenous bacterial populations. Plasmid-mediated bioaugmentation aims to stimulate the spread of contaminant degradation genes among indigenous soil bacteria by the introduction of plasmids, located in donor cells, harboring such genes. But the acquisition of plasmids by recipient cells can affect the host’s fitness, a crucial aspect for the success of plasmid-mediated bioaugmentation. Besides, environmental factors (e.g., soil moisture, temperature, organic matter content) can play important roles for the transfer efficiency of catabolic plasmids, the expression of horizontally acquired genes and, finally, the contaminant degradation activity. For plasmid-mediated bioaugmentation to be reproducible, much more research is needed for a better selection of donor bacterial strains and accompanying plasmids, together with an in-depth understanding of indigenous soil bacterial populations and the environmental conditions that affect plasmid acquisition and the expression and functioning of the catabolic genes of interest.

## Introduction

Soils play a vital role in the provision of ecosystem services and harbor one of the most complex and diverse biological communities on Earth (Barrios, 2007). Therefore, the preservation of soil quality/soil health (both terms are often used interchangeably), defined as “the capacity of soil to perform its ecosystem processes and services, while maintaining ecosystem attributes of ecological relevance” (Garbisu et al., 2011), is currently a matter of great priority. Contamination is one of the most important causes of soil degradation. Only in Europe, there are around 2.5 million potentially contaminated sites, with an annual estimated management cost of 6 billion euros (Panagos et al., 2013). Different anthropogenic activities, such as combustion of fossil fuels, incineration, mining, agricultural practices, urbanization, waste disposal, etc. have contributed to the pressing problem of soil contamination (Besser et al., 2009). Among other negative consequences, the presence of contaminants in soil can cause a negative impact on the soil biota, resulting in an altered activity, biomass and/or diversity of soil biological communities (Burges et al., 2015).

## Remediation of Soil Contaminants

Traditionally, a variety of physicochemical methods (e.g., excavation and disposal in landfills, soil washing, chemical oxidation, encapsulation, thermal treatments, incineration, vitrification, solidification, etc.) have been used for soil remediation. However, these physicochemical strategies are often expensive and, many times, reduce the concentration of soil contaminants at the expense of damaging the integrity of the soil ecosystem (Epelde et al., 2009; Gómez-Sagasti et al., 2016).

The main goal of any soil remediation technology must be not only to reduce the concentration of soil contaminants but to restore soil quality (Epelde et al., 2010; Barrutia et al., 2011; Pardo et al., 2014). A variety of soil physicochemical and biological properties (e.g., parameters that provide information on the biomass, activity and diversity of soil microbial communities) (Epelde et al., 2009; Muñoz-Leoz et al., 2013) are often used as indicators of soil quality. It has also been proposed to assess the effectiveness of remediation methods in terms of the recovery of soil ecosystem services and/or attributes of ecological relevance, such as organization, stability, redundancy, etc. (Garbisu et al., 2011; Epelde et al., 2014).

As an alternative to physicochemical treatments, several biological methods of soil remediation, included within the terms bioremediation and phytoremediation, are currently receiving much attention, mainly owing to their lower cost and environmentally friendly character (Juwarkar et al., 2014). Bioremediation, or the use of microorganisms (bacteria, fungi) to break down contaminants, takes advantage of the catabolic capacity of microorganisms to remove contaminants from soil. However, bioremediation is effective only with a limited range of contaminants and contaminant concentrations. In addition, bioremediation techniques might take too long to achieve the desired reduction in the concentration of soil contaminants (Kumavath and Deverapalli, 2013).

In relation to trace elements (a group of non-degradable contaminants of much concern due to their well-known toxicity), microorganisms can only transform them from one oxidation state or organic complex to another (Garbisu et al., 2002). Then, for the biological remediation of metal contaminated soils, metal-accumulating plants (i.e., accumulators and hyperaccumulators) offer many advantages over microbial processes, as these plants can literally extract the toxic metals from the contaminated site through a phytotechnology termed phytoextraction (Barrutia et al., 2009, 2010; Epelde et al., 2010).

Bioremediation has been successfully employed to remediate soils contaminated with organic contaminants, such as aliphatic hydrocarbons, polycyclic aromatic hydrocarbons, polychlorinated biphenyls, organic solvents and so on (Maphosa et al., 2012).

**FIGURE 1:**
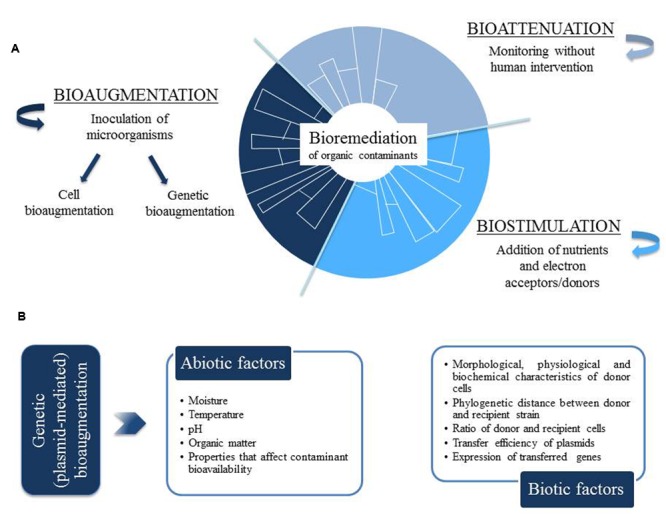
**(A)** Strategies for bioremediation of organic contaminants: bioattenuation, biostimulation and bioaugmentation. **(B)** Environmental factors affecting the efficiency of plasmid-mediated bioaugmentation.

The bioremediation of organic contaminants can be approached by three different strategies: bioattenuation, biostimulation, and bioaugmentation (**Figure 1A**). *Bioattenuation* relies on natural processes to maintain the growth and degrading activity of native microbial populations, so that contaminants are biodegraded without human intervention, apart from the monitoring of contaminant dispersal and degradation rates. Instead, the term *biostimulation* refers to the adjustment of the environmental conditions (e.g., temperature, moisture, aeration, pH, redox potential) and the application of nutrients (e.g., nitrogen, phosphorus) and electron acceptors to contaminated soil, in order to enhance the growth of degrading microbial populations and, then, reduce the concentration of soil contaminants. Finally, *bioaugmentation* has been defined as the inoculation into contaminated soils of microorganisms with the ability to degrade the target contaminants (Maier, 2000; Heinaru et al., 2005). This inoculation can be performed with only one strain or, alternatively, with a consortium of microbial strains with diverse metabolic capacities. The advantage of using a consortium of different strains is that toxic intermediate products generated by one strain may be degraded by another strain (Heinaru et al., 2005). Apart from inoculating wild strains with the required degradation capacities, laboratory-constructed strains with upgraded catabolic abilities have also been considered for a more efficient bioaugmentation (Mrozik et al., 2011).

Iwamoto and Nasu (2001) and El Fantroussi and Agathos (2005) have proposed to apply bioaugmentation in those cases where biostimulation and natural attenuation are proven ineffective. In this regard, in a diesel-contaminated soil, Bento et al. (2005) found bioaugmentation to be more effective for the degradation of the light fraction (C12–C23) of petroleum hydrocarbons than biostimulation. No significant differences were detected between biostimulation and bioaugmentation in relation to the removal of the heavy fraction (C23–C40).

Bioaugmentation can be divided into two different approaches: (i) *cell bioaugmentation*, which relies on the survival and growth of the inoculated strains to perform the degradation of the target contaminants, and (ii) *genetic bioaugmentation*, based on the spread of catabolic genes, located in mobile genetic elements (MGEs), into native microbial populations.

However, despite decades of bioremediation research, the real drivers governing the degradation of organic contaminants are still poorly understood (Meckenstock et al., 2015). In order to gain insight into this question, Meckenstock et al. (2015) revisited and challenged current concepts on the controls and limitations of biodegradation, and pointed out some critical research gaps such as, for instance, the role of protozoa and bacteriophages in shaping communities of bacterial degraders and influencing contaminant degradation rates.

## Cell Bioaugmentation

Cell bioaugmentation is based on the survival and catabolic activity of inoculated microbial strains (Singh and Ward, 2004). The inoculation of bacteria harboring the necessary metabolic pathways for the degradation of the target contaminants can indeed accelerate the removal of such contaminants and, hence, reduce the time required for the intended bioremediation (Nowak and Mrozik, 2016). Inoculated microbial strains must then compete for energy and resources (e.g., nutrients and electron acceptors) with the autochthonous microbial populations already present in the soil ecosystem. The major drawbacks for the successful application of cell bioaugmentation are the (i) frequently very high mortality of the inoculated microbial strains, due to biotic or abiotic stresses, and (ii) limited dispersal of such strains throughout the soil matrix (Pepper et al., 2002; Quan et al., 2010). Many factors, including cell adhesion to soil organic matter (OM), can strongly limit the distribution of bacteria through the soil matrix. To overcome this limitation, several authors (Wang and Mulligan, 2004; Franzetti et al., 2009) have reported the use of surfactants, foams and adhesion-resistant strains.

Despite these limitations, many studies have supported the potential of cell bioaugmentation for the bioremediation of soils contaminated with organic compounds. Wang et al. (2004) reported an accelerated removal of quinoline after the inoculation of *Burkholderia pickettii*. Similarly, Mrozik et al. (2011) showed that cell bioaugmentation with *Pseudomonas* sp. JS150 significantly enhanced phenol degradation in soil, thereby reducing the possibility of formation of phenoxyl radicals (Hanscha et al., 2000). Although the number of *Pseudomonas* sp. JS150 cells decreased significantly during the first few days, the inoculated bacteria were then able to survive over the experimental period and successfully increased the rate of phenol degradation; actually, phenol biodegradation in soil bioaugmented with *Pseudomonas* sp. JS150 cells was 68 and 96 days shorter in clay and sandy soil, respectively, in comparison to non-bioaugmented soil (Mrozik et al., 2011).

## Genetic (Plasmid-Mediated) Bioaugmentation

Genes encoding the degradation of naturally occurring or xenobiotic organic compounds are often located on MGEs, such as plasmids, integrons and transposons. By acquiring these genes through mechanisms of horizontal gene transfer (HGT), recipient bacteria may achieve the capacity to degrade those organic contaminants (Wiedenbeck and Cohan, 2011). HGT allows the exchange of genetic information among bacteria from even distantly related taxonomic groups, thereby allowing bacteria to rapidly adapt to new environmental conditions. Although mutation events can certainly contribute to bacterial adaptation, mutation rates in bacterial populations are generally low. Besides, it is currently assumed that an increased rate of mutations would result in increased death owing to deleterious effects (Martínez et al., 2009).

Out of the three mechanisms of HGT in bacteria (i.e., transformation, transduction and conjugation), conjugation is a most efficient biological process in which genetic information encoded in plasmids is transferred, from donor to recipient bacteria, by direct cell-to-cell contact (Furuya and Lowy, 2006). Bacterial conjugation is known to accelerate the dissemination of resistance to, for instance, antibiotics and heavy metals, as well as to facilitate the distribution of genes involved in the degradation of organic compounds. Nevertheless, the contribution of conjugation to HGT among soil bacteria and the factors involved in the transfer and proliferation of plasmid-containing bacteria in the soil ecosystem are yet not fully understood.

Bacterial adaptation through evolutionary time has been shaped, among other aspects, by the high plasticity of bacterial genomes, which allows bacteria to rearrange and exchange genomic sequences, thus opening the possibility to acquire beneficial traits (Sørensen et al., 2005). As a matter of fact, the loss, rearrangement and acquisition of functional genetic modules can have a vast impact on the extent and speed of the evolutionary adaptation of bacteria (Wozniak and Waldor, 2010; Bertels and Rainey, 2011). MGEs are, to a great extent, responsible for these processes of gene mobility and reorganization, both within genomes (intracellular) and between bacterial cells (intercellular).

Many of the studies on lateral dissemination of genetic material among bacteria have focused on antibiotic and metal resistance. Research on the horizontal transfer of genes associated with the degradation of organic compounds in natural environments, such as the soil ecosystem, is still insufficient to fully understand the mechanisms involved in such process (Christensen et al., 1998; Top et al., 1998; Dejonghe et al., 2000; Aspray et al., 2005; Overhage et al., 2005; Musovic et al., 2010). In any case, some plasmids, such as those implicated in the catabolic pathway of 2,4-dichlorophenoxyacetic acid (2,4-D), have been thoroughly studied (Top et al., 1998; Dejonghe et al., 2000; Newby and Pepper, 2002).

Plasmid transfer between soil bacteria has been contemplated as a promising strategy for the dissemination of catabolic functions within soil bacterial communities (Venkata Mohan et al., 2009; Mrozik and Piotrowska-Seget, 2010). As abovementioned, plasmid-encoded metabolic pathways can be transferred among bacteria, thus playing a critical role in the adaptation of bacteria to different environmental conditions (Reineke, 1998; Sayler and Ripp, 2000). Specifically, HGT has been reported to promote bacterial adaptation to the presence of organic contaminants (Top and Springael, 2003).

The underlying idea behind genetic (plasmid-mediated) bioaugmentation is to stimulate the rate of contaminant degradation by increasing, through HGT, the number and diversity of native bacteria with the capacity to metabolize the target contaminants. In this respect, it must be emphasized that numerous catabolic pathways involved in the degradation of organic contaminants have been identified in MGEs (Top et al., 2002; Jussila et al., 2007).

Genetic (plasmid-mediated) bioaugmentation is defined as a technology in which donor bacteria harboring self-transmissible catabolic plasmids are introduced into the soil matrix in order to enhance, by HGT, the potential and rate of contaminant degradation of existing bacterial populations (Top et al., 2002; Ikuma and Gunsch, 2010, 2012). Compared to cell bioaugmentation, plasmid-mediated bioaugmentation appears *a priori* a more effective strategy for the bioremediation of organic contaminants, as the bacteria that will eventually degrade the contaminants (i.e., bacteria with the recently acquired plasmids harboring the necessary catabolic genes) are expected to be adapted to live in the soil under remediation. In this manner, one of the main drawbacks for the successful application of cell bioaugmentation, i.e., the low survival of the inoculated microbial strains, appears to be overcome.

For plasmid-mediated bioaugmentation, both an appropriate selection of donor bacteria with the required plasmid and a profound knowledge of native soil bacterial populations are required to increase the probability of an efficient plasmid acquisition and the expression of the catabolic genes of interest.

Many studies on plasmid-mediated bioaugmentation have been published (**Table 1**). In a microcosm study, Halden et al. (1999) detected an enhanced degradation of 3-phenoxybenzoic acid (3-POB) as a result of the transfer of plasmids pPOB and pD30.9 from *Pseudomonas pseudoalcaligenes* POB310 (pPOB) and *Pseudomonas* sp. B13-D5 (pD30.9) and B13ST1 (pPOB) to recipient soil bacteria. Using *P. putida* as donor strain of two catabolic plasmids (pEMT1 and pJP4), Dejonghe et al. (2000) reported the degradation of 2,4-D in soil under microcosm conditions. These authors investigated the bioaugmentation potential of plasmids pEMT1 and pJP4 in two soil layers (0–30 and 30–60 cm soil depth) differing in physicochemical properties and microbial community structure, finding out a more efficient degradation of 2,4-D in the deeper soil layer where the indigenous microbial communities lacked the ability to catabolize 2,4-D. Under microcosm conditions, Inoue et al. (2012) studied the effect of bioaugmentation with *P. putida* and *Escherichia coli* cells, harboring the self-transmissible 2,4-D degradative plasmid pJP4, on the degradation of 2,4-D. These authors found that the number of *P. putida* and *E. coli* cells decreased rapidly after their inoculation in a 2,4-D contaminated soil slurry, but the degradation of this contaminant was nevertheless stimulated, most likely due to the occurrence of transconjugants resulting from the transfer of plasmid pJP4. Inoue et al. (2012) concluded that genetic bioaugmentation with *P. putida* and *E. coli* cells harboring plasmid pJP4 can stimulate the degradation of 2,4-D in soil without a substantial impact on the soil microbial community, as reflected by the values of parameters which provide information on carbon source utilization (through the use of the well-known Biolog^TM^ plates) and nitrogen transformations (nitrate reduction assay, quantification of amoA gene of ammonia-oxidizing bacteria, quantification of *nirK* and *nirS* genes of denitrifying bacteria). In sequencing batch reactors, Tsutsui et al. (2013) achieved a complete degradation of 2,4-D by plasmid (pJP4)-mediated bioaugmentation with *Cupriavidus necator* JMP134 and *E*. *coli* HB101 as donor strains. These authors were able to identify the emergence of 2,4-D-degrading transconjugants associated to *Achromobacter, Burkholderia, Cupriavidus* and *Pandoraea.*

**Table 1:** Examples of plasmid-mediated bioaugmentation studies.

Contaminant	Plasmid	Strain	Reference
3-phenoxybenzoic acid	pPOB	*P. pseudoalcaligenes POB310*	Halden et al., 1999
3-chlorobenzoate	pBRC60	*C. testosteroni*	Pepper et al., 2002
2,4-dichlorophenoxyacetic acid	pJP4	*C. necator* JMP134 *E. coli* HB101	Tsutsui et al., 2013
2,4-dichlorophenoxyacetic acid	pJP4	*P. putida* KT2442 *E. coli* HB101	Inoue et al., 2012
2,4-dichlorophenoxyacetic acid	pEMT1 pJP4	*P. putida* UWC3	Dejonghe et al., 2000
2,4-dichlorophenoxyacetic acid	pJP4	*R. eutropha* JMP134 *E. coli* D11	Newby et al., 2000
γ-hexachlorocyclohexane	pLB1	*S. japonicum* UT26DB	Miyazaki et al., 2006
Chlorpyrifos	pDOC	*E. coli* JM109	Zhang et al., 2012
Dichlorodiphenyltrichloroethane	pDOD	*E. coli* TG I	Gao et al., 2015
Naphthalene	pNF142	*P. putida* BS394 *P. putida* KT2442	Filonov et al., 2010
Oil	pWW0-derivative TOL	*P. putida* PaW85	Jussila et al., 2007
Toluene	pWW0-derivative TOL	*P. putida* BBC443	Ikuma and Gunsch, 2012

Pepper et al. (2002) conducted microcosm experiments to enhance the degradation of 3-chlorobenzoate (3-CB) using plasmid pBRC60, which harbors genes for 3-CB mineralization, and *Comamonas testosteroni* as donor strain. Although they did observe degradation of 3-CB, they could not detect any transfer event of plasmid pBRC60 from *C. testosteroni* to native soil bacteria.

Miyazaki et al. (2006) isolated a plasmid (pLB1) involved in the dissemination of genes for γ-hexachlorocyclohexane (lindane) degradation. This plasmid, carrying the *linB* gene, was isolated from *Sphingobium japonicum* UT26DB and then successfully transferred, under laboratory conditions, from this strain to other α-proteobacterial strains but not to any of the β- or γ-proteobacterial strains tested.

In their study on the transfer of TOL plasmid (also designated pWW0) during bacterial conjugation *in vitro* and rhizoremediation of oil-contaminated soil *in vivo*, Jussila et al. (2007) demonstrated the successful transfer of TOL plasmid for toluene degradation from *P. putida* PaW85 to *P. oryzihabitans* 29. In rhizosphere microcosms, Mølbak et al. (2007) found that the transfer of plasmid pWW0 from *P. putida* resulted in transconjugants belonging to *Enterobacteria* and *Pseudomonas*. This well-characterized self-transmissible catabolic plasmid, pWW0, was also used by Ikuma and Gunsch (2012) to assess its potential for bioaugmentation in toluene-contaminated soil slurry.

Under laboratory conditions, horizontal transfer of plasmid pGKT2 was successfully carried out by Jung et al. (2011) from *Gordonia* sp. KTR9 to *Gordonia polyisoprenivorans, Rhodococcus jostii* RHA1 and *Nocardia* sp. TW2 strains. These transconjugants showed the ability to use hexahydro-1,3,5-trinitro-1,3,5,-triazine (RDX) as a nitrogen source.

In a contaminated field site located in Cixi, Zhejiang (China), Gao et al. (2015) achieved effective plasmid-mediated bioaugmentation for the degradation of dichlorodiphenyltrichloroethane (DDT) in soil with *E. coli TG I* (pDOD-gfp) as donor strain. In this study, the catabolic plasmid pDOD from *Sphingobacterium* sp. D-6 was conjugally transferred to soil bacteria, such as members of *Cellulomonas*, and accelerated DDT degradation. Different studies have reported the use of the GFP (green fluorescence protein) detection system to monitor plasmid transfer from donor cells to indigenous soil bacteria in soil slurries (Ikuma et al., 2012) and field contaminated soil (Gao et al., 2015).

Filonov et al. (2010) determined transfer frequencies in open soil after inoculation with genetically tagged plasmid-containing naphthalene-degrading *P. putida* KT2442 and auxotrophic donor BS394 (pNF142::Tn*Mod*-OTc) cells, finding out that plasmid pNF142 was transferred to native soil bacteria (mainly to fluorescent pseudomonads) at a frequency of 4 × 10^-6^/donor cell. After bioaugmentation with *E. coli* JM109 (pDOC-gfp) strain, Zhang et al. (2012) observed that pDOC plasmid was transferred to native soil bacteria under microcosm conditions, including members of *Pseudomonas* and *Staphylococcus* which acquired the capacity to degrade chlorpyrifos (a widely used insecticide). As it is usually the case, the efficiency of this transfer, as measured by the chlorpyrifos degradation efficiency and the number of chlorpyrifos degraders, was influenced by soil type, temperature and moisture content (Zhang et al., 2012).

Finally, attention has also been paid to the use of genetically modified organisms (GMOs) for bioaugmentation. Nonetheless, the deliberate release of GMOs into the environment is subjected to regulatory constraints (Garbisu and Alkorta, 1997; Sayler and Ripp, 2000; Directive 2001/18/EC, 2001). The transfer of catabolic genes between GMOs and wild bacterial strains might facilitate the acquisition and spread of new degradative pathways among indigenous bacterial communities. To this purpose, Massa et al. (2009) engineered the recombinant strain *P. putida* PaW340/pDH5, constructed by cloning dehalogenase genes from *Arthrobacter* sp. FG1 in *P. putida* PaW340, for the degradation of 4-chlorobenzoic acid (CBA) in soil slurry. After inoculation of this recombinant strain into soil slurry, a higher degradation of CBA was observed, compared to the slurry inoculated with pre-adapted cultures of *Arthrobacter* sp. FG1.

## Effect of Plasmid Acquisition On Host Fitness

The success of plasmid-mediated bioaugmentation for the bioremediation of contaminated soil relies not only on an efficient transfer of the required plasmid from donor bacteria to soil recipient bacteria, but also on the ability of recipient cells to properly express the plasmid-harbored catabolic genes, so that the desired phenotypic changes (i.e., biodegradation of the target contaminant) can be attained. After plasmid acquisition, the capacity of recipient cells to successfully perform the desired catabolic function depends, among other factors, on their newly acquired competitive abilities and on the alteration of the host’s own competitive abilities (van Rensburg et al., 2012). A thorough understanding of how plasmid acquisition can affect host fitness is fundamental to then achieve the persistence of the introduced plasmid in the recipient cells.

Plasmid acquisition can provide recipient bacteria with a large array of beneficial traits, such as catabolic potential, resistance to antibiotics and/or metals, faster growth, ability to use a wider range of compounds as energy sources, etc. (Top et al., 1998; Riley and Wertz, 2002). In many cases, plasmid-harboring hosts have been found to be competitively fitter than their plasmid-free counterparts (Dionisio et al., 2005; Starikova et al., 2013). Nonetheless, horizontally acquired genes can also function inefficiently in the genomic background of recipient cells (Chou et al., 2011; Park and Zhang, 2012). After all, horizontally acquired genes find themselves immersed in a new metabolic context and their function relies on the host’s machinery. Genetic determinants often encounter the required metabolic “partners” (e.g., substrates, proteins) in the recipient cells, so that the intended changes in the host’s metabolism become possible. Conversely, other times, the required metabolic partners for the proper functioning and regulation of newly acquired genes are missing in the recipient cells. Indeed, the acquisition of plasmids can negatively affect cellular networks in recipient cells and, concomitantly, trigger fitness costs as collateral damage (Bouma and Lenski, 1988; Martínez et al., 2009). Fitness (metabolic) costs derived from plasmid acquisition can be highly variable (De Gelder et al., 2007), as they can originate from a variety of factors, including: (i) *energetic costs* due to consumption of molecular building blocks and/or energy sources derived from the activity of horizontally acquired regions; (ii) *chromosomal disruption* by horizontally acquired genes, when such genes are incorporated into the chromosome; (iii) *sequestration of cellular processes and associated molecular machinery* (e.g., ribosomes) by the horizontally acquired regions; and (iv) *plasmid size*, since small plasmids can carry only a single accessory determinant but large plasmids can carry more than 10 accessory determinants as well as other genes (Shachrai et al., 2010; Baltrus, 2013; Vogwill and MacLean, 2015).

Fitness costs associated to plasmid acquisition can be offset by benefits derived from the fact that plasmids are ideal biological tools to create genetic variation within bacterial populations. A major benefit from maintaining transferable plasmids derives from the fact that, in this manner, bacterial populations can gain stability against potential environmental changes.

Bacteria with acquired genes can, on the other hand, alleviate fitness costs through compensatory evolution (San Millan et al., 2014). Thus, for instance, bacteria can minimize plasmid-related fitness costs by integrating only the desired plasmid-acquired determinants in the chromosome.

Conjugative plasmids (i) are usually large (they encode genes for the conjugation process itself and for stabilization within the host); (ii) are normally found in low copy number; (iii) appear well maintained over successive generations (Norman et al., 2009; Jung et al., 2011); and (iv) act as fundamental vehicles of HGT (Frost et al., 2005; Thomas and Nielsen, 2005).

In their laboratory study on the capacity of *Gordonia* sp. KTR9 to transfer plasmid pGKT2 and the associated RDX (hexahydro-1,3,5-trinitro-1,3,5,-triazine) degradation ability to other bacteria, Jung et al. (2011) investigated plasmid stability after HGT from *Gordonia* sp. KTR9 to *G. polyisoprenivorans, R. jostii* RHA1 and *Nocardia* sp. TW2, finding out a marked decrease in plasmid retention after 50 generations with *Nocardia* sp. TW2, while *G. polyisoprenivorans* and *R. jostii* RHA1 transconjugants exhibited retention of pGKT2 plasmid for 100 generations. It was speculated that this decreased stability in *Nocardia* sp. TW2 might have been caused by a larger metabolic expense incurred by the incorporation of pGKT2 in this strain, compared to the other two bacterial strains (Jung et al., 2011).

Given that positive selection cannot explain the long-term stability of costly plasmids (Hall et al., 2017), the explanation for such long-term stability remains a most challenging task, since segregational loss and the cost of plasmid carriage should drive the loss of plasmids through purifying selection (Hall et al., 2017). In this respect, two evolutionary routes to plasmid stability appear possible (Hall et al., 2017): (i) the evolution of high conjugation rates would allow plasmids to survive as infectious agents through horizontal transmission (Hall et al., 2016; Kottara et al., 2016); and (ii) compensatory evolution to ameliorate the cost of plasmid carriage can weaken purifying selection against the plasmid backbone (Harrison et al., 2015; Porse et al., 2016).

Finally, it must be taken into consideration that plasmids can be classified into incompatibility groups (incompatibility defined as the inability of plasmids sharing similar replication and partition systems to be propagated stably in the same host cell line; in other words, members of each group cannot co-reside within the same bacterial host), such as IncP, IncN, IncW, and IncF. Incompatibility groups have been independently classified in three different genera: there are 27 Inc groups in *Enterobacteriaceae*, 14 Inc groups in *Pseudomonas*, and approximately 18 Inc groups in *Staphylococcus* (Shintani et al., 2015). Plasmids classified in *E. coli* as IncP and in *Pseudomonas* as IncP-1 are a well-studied group of plasmids that can carry a variety of phenotypic markers, including antibiotic resistance, metal resistance and the ability to degrade xenobiotics. It has been reported (Popowska and Krawczyk-Balska, 2013) that a detailed analysis of IncP-1 plasmid genomes could provide useful information for the development of effective methods of soil bioremediation. After all, the evolutionary adaptation of microorganisms to the presence and utilization of organic contaminants is often due to plasmids (mainly, from IncP group) that carry genes encoding enzymes involved in the degradation of those compounds. For instance, plasmids IncP-1, IncP-7 and IncP-9 contain genes encoding enzymes required for the degradation of naphthalene, toluene, chlorobenzene, p-toluenesulfonate, 2,4-D, haloacetate and atrazine (Shintani et al., 2010a,b; Popowska and Krawczyk-Balska, 2013). Relevantly, there seems to be a distinction between (i) plasmids that harbor genes for the degradation of naturally occurring compounds and (ii) plasmids that harbor genes for the degradation of xenobiotics (Top et al., 2002): degradation of naturally occurring compounds is often encoded in IncP-2 and IncP-9 plasmids, while the degradation of xenobiotics seems to be encoded by the well-known broad host range IncP-1 plasmids. IncP-1 plasmids are very promiscuous, and this promiscuity appears to play a crucial role in the evolution of new metabolic pathways by recruiting catabolic genes or gene segments from different organisms into a suitable host (Wyndham et al., 1994; Beil et al., 1999).

Therefore, different plasmids potentially useful for plasmid-mediated bioaugmentation (with, for instance, each plasmid harboring a gene encoding a different enzyme involved in the degradation route of a specific contaminant) cannot co-reside within the same host if they belong to the same incompatibility group. Then, if we want to apply different plasmids from the same incompatibility group, each of them harboring a gene for a specific step in the contaminant degradation pathway, they must be applied in different donor cells and, for an effective biodegradation, each plasmid should be transferred to a different recipient cell, decreasing considerably the probability of successful plasmid-mediated bioaugmentation.

## Influence of Abiotic and Biotic Factors On Bioaugmentation

The success of both cell and plasmid-mediated bioaugmentation greatly depends on the environmental (abiotic and biotic) conditions present in the soil to be remediated (Cho et al., 2000; Bento et al., 2005; Wolski et al., 2006). In fact, during plasmid-mediated bioaugmentation, environmental factors can play important roles in the (i) transfer efficiency of catabolic plasmids, (ii) expression of horizontally acquired genes and, finally, (iii) contaminant degradation activity (Popa et al., 2011; Ikuma and Gunsch, 2012). In particular, several abiotic factors such as soil moisture, temperature and OM content are known to affect bioaugmentation efficiency (**Figure 1B**).

Soil moisture can have an effect on plasmid transfer during plasmid-mediated bioaugmentation by affecting the contact between donor and recipient bacteria (Miller et al., 2004; Aminov, 2011). In this respect, Gao et al. (2015) evaluated the effectiveness of plasmid-mediated bioaugmentation for *p,p′*-DDT degradation at three different soil moisture conditions (40, 60, and 80%), and concluded that 60% moisture content was optimal for maximum plasmid transfer efficiency.

Temperature has been shown to affect plasmid transfer efficiency (Inoue et al., 2005; Zhang et al., 2012). For the enhancement of DDT degradation by plasmid-mediated bioaugmentation with plasmid pDOD, the optimal temperature interval for cell growth and activity of both donor and recipient soil bacteria was established at 25–30°C (Gao et al., 2015). Johnsen and Kroer (2007) found that increasing temperatures resulted in an increase in the transfer of plasmid pRO103 encoding resistance to mercury and tetracycline and partial degradation of 2,4-D.

Regarding soil OM content, in a bioaugmentation laboratory experiment, Greer and Shelton (1992) observed higher rates of mineralization of 2,4-D in soil with a low OM content, compared to soil with a high content of OM. Under laboratory conditions, Kim et al. (2008) found that *P. spadix* BD-a59 cells were able to degrade BTEX at a slower rate in soil with low OM content than in organic-rich soil. When studying the biodegradation of polychlorinated biphenyls (PCB) in soil under laboratory conditions, Haluška et al. (1995) observed that humic acids affected the survival and activity of the inoculated *Alcaligenes xylosoxidans* strain, which exhibited maximum survival rates in soil with an intermediate amount of organic carbon and the highest amount of aromatic carbon in humic acids. Highest levels of PCB degradation were found in soil with the highest content of organic carbon and an intermediate amount of aromatic carbon in humic acids (Haluška et al., 1995).

Wang et al. (2014) performed plasmid transfer experiments between soil bacteria, using a TOL-like plasmid carrying the gene encoding for catechol 2,3-dioxygenase, to study some factors (soil depth, soil type, etc.) that could affect the transfer of plasmids, finding out that these factors certainly have a considerable effect on the transfer of the TOL-like plasmid in soil. Concerning soil depth, under microcosm conditions, Wang et al. (2014) found, in general, lower frequencies of plasmid transfer at greater soil depths, a fact most likely due to the often-found gradual decrease in bacterial biomass and activity at increasing soil depths, possibly related to concomitantly decreased oxygen concentrations (Król et al., 2011). Król et al. (2011) reported that oxygen concentration can affect plasmid transfer through an oxygen-related mechanism or indirectly via its impact on cell physiology. When studying the influence of soil type (loamy sand, sandy loam, sandy clay loam, loam) on plasmid transfer, Wang et al. (2014) observed a highest frequency of plasmid transfer in loam soil, probably related to the fact that loam often contains more nutrients and humus than other soil types, and higher values of microbial biomass and metabolic activity (Djokic et al., 2013).

In the same way, the chemical nature, concentration and bioavailability of the contaminants are crucial factors influencing bioaugmentation efficiency (Davis and Madsen, 1996; Stalwood et al., 2005). Sejáková et al. (2009) reported a relationship between pentachlorophenol (PCP) concentration in soil and the number of CFU of the *C. testosteroni* CCM7530 strain used for bioaugmentation: at a PCP concentration of 100 mg kg^-1^, the number of *C. testosteroni* CCM7530 CFUs rapidly increased over 17 days, while, at 10 mg PCP kg^-1^, the number of CFUs initially decreased until day 7 to then increase until day 17.

In any case, the level of selective pressure required to promote conjugal plasmid transfer depends on the specific contaminant and its concentration, as well as on the specific catabolic plasmid. In soil slurry, Ikuma and Gunsch (2012) observed that environmentally relevant concentrations of toluene might not exert enough pressure for transfer of plasmid TOL from *P. putida* BBC443 to *Serratia marcescens* and *P. fluorescens* cells. In their study on the degradation of 2,4-D, DiGiovanni et al. (1996) observed that this contaminant originated the required selective pressure for conjugal transfer of the intended catabolic plasmids.

Many biotic factors can also affect the success of plasmid-mediated bioaugmentation. Some genetic differences, such as guanine-cytosine (G+C) content and phylogenetic relationship between donor and recipient strain, can negatively affect the expression of the catabolic phenotype following conjugal plasmid transfer, as described by Ikuma and Gunsch (2012). Indeed, for plasmid-mediated bioaugmentation, biological differences between donor and recipient bacterial strains such as, for example, phylogenetic distance (Popa et al., 2011) and plasmid host range (De Gelder et al., 2005; Sorek et al., 2007), can play an important role. In 2,4-D contaminated soils, Newby et al. (2000) studied the bioaugmentation efficiency of two plasmid pJP4-bearing bacteria (the natural host, *Ralstonia eutropha* JMP134, and a laboratory-generated *E. coli* strain amenable to donor counterselection, named *E. coli* D11) and concluded that the correct choice of donor strain is a factor of the utmost importance for bioaugmentation.

Ikuma and Gunsch (2012) indicated that the success of plasmid-mediated bioaugmentation is dependent on: (i) high transfer rates of the catabolic plasmid to as many indigenous bacteria as possible; and (ii) the high expression level of an active contaminant-degrading phenotype in all transconjugants following conjugal plasmid transfer. Then, prior to the bioaugmentation process itself, it is important to characterize potentially recipient soil bacterial communities, paying special attention to dominant taxonomic groups. In the last years, next generation sequencing has provided a more comprehensive analysis of indigenous soil bacterial communities (Walsh, 2000), opening the door to the identification of potential recipient bacterial populations, and therefore a more informed selection of both the donor strain and the plasmid type (Ikuma and Gunsch, 2012).

Other biotic factors, such as competition between inoculated and indigenous bacteria for carbon sources, antagonistic interactions and predation by protozoa and bacteriophages, etc. also play an essential role in bioaugmentation efficiency. The critical factor is the selection of the right bacterial strains (Thompson et al., 2005), since the inoculated strain must be able not only to degrade the target contaminant (or, in the case of plasmid-mediated bioaugmentation, to be able to effectively transfer the catabolic plasmid), but also to successfully compete with indigenous microbial populations and, in general, soil biota. On the other hand, plasmid transfer frequency has been shown to depend on the initial cell density ratio between donor and recipient cells (Pinedo and Smets, 2005; Ikuma et al., 2012).

Morphological, physiological and biochemical characteristics such as, for instance, cell size, growth rate, resource utilization ability, resistance phenotypes, biofilm formation capacity, cell motility, etc. are key traits for bacterial survival and competitiveness. Furthermore, DNA content has a marked influence on bacterial ecophysiological traits (i.e., adaptive traits to environmental changes) affecting, among other aspects, the rate of cell growth (Wickham and Lynn, 1990). Nevertheless, despite the assumption that fitness costs associated to HGT are caused by the need to maintain and replicate the extra-DNA, some studies indicate that they are predominantly due to transcription and translation processes (Bragg and Wagner, 2009; Shachrai et al., 2010).

The capacity of the host to use different carbon substrates before and after plasmid acquisition can provide an estimation of (i) its competitive ability and (ii) changes specifically associated to the plasmid transfer itself. Biolog^TM^ plates can be employed to obtain a phenotypic fingerprint of bacterial strains in relation to their capacity to use a variety of carbon sources. Karve et al. (2016) followed phenotypic variations, using Biolog GEN III MicroPlates^TM^, to assess functionally relevant consequences of DNA changes.

Antibiotic resistance is probably the most extensively studied bacterial competitive trait. As a consequence of the production of antibiotics by soil microbial populations (D’Costa et al., 2006), soil is thought to be the largest reservoir of antibiotic resistance genes. Owing to fitness costs associated to antibiotic resistance, when bacteria change to an antibiotic-free environment, resistance is expected to disappear (Morosini et al., 2000), according to the assumption that, in the absence of selective pressure, resistant bacteria with a lower fitness will be outcompeted by susceptible counterparts with a higher fitness. However, it seems that bacteria tend to keep the mechanisms of antibiotic resistance, in order to maintain such an advantageous trait in the face of a possible change in environmental conditions (San Millan et al., 2014). Besides, in nature, antibiotics and antibiotic resistance determinants might play a variety of roles (e.g., signaling molecules in quorum sensing and biofilm formation, production of virulence factors, host-parasite interactions) (Sengupta et al., 2013) that justify the preservation of antibiotic resistance determinants in the absence of the selective pressure.

Biofilms are known to protect bacterial cells against antimicrobials (Høiby et al., 2010), predation, oxidative stress (Geier et al., 2008), etc. Biofilms harbor spatially structured bacterial communities where plasmids can be more easily shared through HGT (Jefferson, 2004), facilitating, for instance, the dissemination of catabolic genes. Remarkably, attachment to surfaces by biofilm-associated factors is another cellular function associated to genes present in plasmids (Norman et al., 2009).

Cell motility is a critical aspect for the necessary dispersal of inoculated bacteria toward the target contaminants. Nevertheless, although highly motile bacterial cells, in their search for energy and nutrients, can disperse more easily into the surrounding environment, they also have a higher probability of encountering potential competitors (Reichenbach et al., 2007). In any case, motile bacterial populations, such as swarming bacteria, can more rapidly colonize new niches, with the associated ecological benefits (Verstraeten et al., 2008). Interestingly, there is a complex link between motility and biofilm formation because both processes appear to involve similar components at certain stages and conditions (Verstraeten et al., 2008).

(Gardin and Pauss, 2001; Gentili et al., 2006) have used different strategies of cell encapsulation and immobilization to facilitate inoculation survival, by providing a protective niche and temporary nutrition for the inoculated bacteria. Carrier materials, such as charcoal (Beck, 1991), nylon (Heitkamp and Steward, 1996), chitin, chitosan (Gentili et al., 2006; Chen et al., 2007) and zeolite (Liang et al., 2009) have been used in an attempt to maintain inoculant activity over a sufficiently long period of time after strain inoculation.

It must be taken into consideration that the influence of all these abovementioned abiotic and biotic factors has only been studied in a very limited number of bacterial strains and, in many cases, under controlled simplified environmental conditions, very different from those encountered in the natural environment. Therefore, many more in-depth studies on the impact of abiotic and biotic factors on cell and plasmid-mediated bioaugmentation are needed.

## Concluding Remarks

Both cell bioaugmentation and genetic (plasmid-mediated) bioaugmentation have proven effective for the bioremediation of soils contaminated with organic compounds. However, cell bioaugmentation has an important limitation, i.e., the frequently very high mortality of the inoculated microbial strains, due to biotic or abiotic stresses. Then, *a priori*, plasmid-mediated bioaugmentation appears to have greater potential than cell bioaugmentation, since plasmids can act as gene-messenger biological tools that can transfer the required catabolic genes to indigenous bacterial populations already adapted to the soil under remediation. But for plasmid-mediated bioaugmentation to be successful and reproducible, much more research is needed for a better selection of donor bacterial strains and accompanying plasmids, together with an in-depth understanding of indigenous soil bacterial populations and the environmental conditions that affect plasmid acquisition and the expression and functioning of the catabolic genes of interest. Similarly, further research is required to better understand and then improve the ecological fitness of recipient bacterial strains in the contaminated soil.

## Author Contributions

CG and IA: Design of the work and the acquisition of the data, writing and revision of the content, approval of the last version and ensuring accuracy and integrity of the work. LE and OG: Acquisition of the data, writing and revision of the content, approval of the last version of the work. EG: Writing and revision of the content, approval of the last version and ensuring accuracy and integrity of the work.

## Conflict of Interest Statement

The authors declare that the research was conducted in the absence of any commercial or financial relationships that could be construed as a potential conflict of interest.
